# “If I was with my father such discrimination wouldn’t exist, I could be happy like other people”: a qualitative analysis of stigma among peacekeeper fathered children in the Democratic Republic of Congo

**DOI:** 10.1186/s13031-020-00320-x

**Published:** 2020-11-13

**Authors:** Kirstin Wagner, Heide Glaesmer, Susan A. Bartels, Sabine Lee

**Affiliations:** 1 Department of History, University of Birmingham, Birmingham, UK; 2 Department of Medical Psychology and Medical Sociology, University of Leipzig, Leipzig, Germany; 3 Departments of Emergency Medicine and Public Health Sciences, Queen’s University, Kingston, Canada

**Keywords:** Stigma, Children Born of War, United Nations, Peacekeeping, Sexual Misconduct, Democratic Republic of Congo, Transgenerational

## Abstract

**Background:**

The United Nations (UN) Organization Stabilization Mission in the Democratic Republic of Congo (MONUSCO) comprises the largest and longest serving peacekeeping operation to date. Since the launch of the mission in 1999, sexual relations between UN peacekeepers and the local population regularly occur; some resulting in children being conceived. Reports have indicated that women and girls bearing children from such relations face difficult socio-economic realities. The present study is the first to explore the situation of peacekeeper fathered children (PKFC) through a qualitative analysis that includes interview material from mothers and child participants.

**Methods:**

The article uses theories from stigma research to illustrate how children conceived through sexual relations with UN peacekeepers integrate into social networks. We conducted a case study of mothers and their PKFC at different sites of UN peacekeeping (UNPK) in eastern Democratic Republic of Congo (DRC). Based on 95 in-depth interviews held in 2018, a thematic qualitative analysis examined experiences of stigma and discrimination. In order to understand the challenges of mothers and children from a transgenerational perspective, we evaluated perceptions of rejection rooted in the mother-child relationship.

**Results:**

Of the mothers and children surveyed, a large majority struggled with stigmatizing behaviour by family and/or community members. PKFC perceived their discrimination to be based upon their mixed ethnicity, fatherlessness, illegitimacy at birth, as well as a lack of resources and opportunity. Mothers most often attributed their stigma to economic deprivation, extra-marital sexual relations, single parenting and being associated with UNPK. Parallels in the experiences of mothers and children suggest a bi-directional transmission of status loss and stigma between generations.

**Conclusions:**

This is the first empirical study to compare the situation of PKFC and their mothers in any country of UNPK deployment. The findings highlight multiple burdens that affect their daily lives and illustrate an interplay between drivers of stigmatization for mothers and children. The overarching needs identified are financial, and these call for action regarding policies and programmes that provide resources to those concerned. The results further demonstrate the need for psychosocial support that considers transgenerational dynamics and both mothers and children as core addressees of assistance.

## Background

The armed conflict in the Democratic Republic of Congo (DRC) has been described as the deadliest conflict since World War II [1]. Despite perceptible improvements in the past two decades, the country continues to suffer from recurring cycles of violence and a distressing amount of sexual and gender-based violence (SGBV) [2, 3]. In response to the ongoing instability, the United Nations (UN) Security Council (UNSC) established the UN Organization Mission in the DRC (MONUC) in 1999 [4]. Renamed to the UN Stabilization Organization Mission in 2010 (MONUSCO), peacekeepers were authorized to use force in order to protect civilians against the multiple foreign and domestic armed groups [5]. In 2013, the UNSC introduced an “intervention brigade” with the aim to target the security vacuum and aggravating humanitarian crises in the east [6]. As of December 2019, 17.000 personnel are deployed in DRC [7]. Despite peacekeepers being mandated to protect civilians, their daily presence for over 20 years has come with challenges for the local population. Sparked by their prolonged proximity to one another, sexual relations (e.g. rape, business arrangements, romantic relationships) between UN peacekeeping (UNPK) personnel and local women and girls emerged [8].

Allegations of sexual misconduct during UNPK have unfolded from a long list of peacekeeping operations (PKO), still, only a small fraction of impeached peacekeepers are prosecuted or convicted [8, 9]. Irrespective of the UN strongly discouraging all sexual contact between peacekeepers and residents in host countries [10], sexual encounters regularly occur; some with far-reaching consequences. Reports indicate that women in different geopolitical settings have children fathered by members of UNPK forces [11, 12], yet to date, little is known about their experiences. According to estimates from the Geneva Centre for the Democratic Control of Armed Forces, approximately 25.000 children were left behind by peacekeepers in Cambodia and another 6.600 in Liberia [13]. The first systematic study addressing children left behind by peacekeepers confirmed in 2019 that during the UN Stabilization Mission in Haiti (MINUSTAH), a sizeable group of “pitit MINUSTAHs” (little MINUSTAHs) or “bébés casques bleus” (blue helmet babies) were born [14]. Anecdotal reports from the DRC suggest that children abandoned by UNPK staff are not uncommon (e.g., [15–18]), however, no comprehensive statistics exist.

Euphemistically referred to as “peace-babies”, children fathered by UN peacekeepers (hereafter PKFC) are considered a subgroup of children born of war (CBOW) [19]. The term CBOW refers to individuals with one parent who is part of a foreign army or peacekeeping force and another who belongs to the local population in the country of deployment [20, 21]. Neglected for a long time, the experiences of CBOW have recently become a focus of academic study. A conceptual framework defining the psychosocial consequences of growing up as a CBOW was developed in 2012 [22]. Glaesmer et al. emphasize three aspects affecting the mental health of CBOW: experiences of stigmatization and discrimination [23, 24], child maltreatment [25], and identity formation [26]. While all three categories pose substantial challenges to CBOW [27, 28], this article focuses on stigmatization because it was the major concern of those interviewed in the present study.

Stigma, according to Goffman’s seminal work [29, 30], is a “deeply discrediting attribute” which reduces an individual “from a whole usual person to a tainted, discounted one”. The focus of psychological discourses has shifted to a less individualistic view, emphasizing that stigma is socially constructed within a certain context [31, 32]. The widely used sociological definition, by Link and Phelan [33], combines different stigma concepts (labelling, stereotyping, separation, status loss and discrimination) and highlights power dynamics that allow it to flourish. Subsequent frameworks emphasized structural determinants such as economic, political or historical factors that enable stigma to translate into social injustice [34, 35]. Cross-cultural research suggests that stigma experiences are universal, but that the process of being discredited affects individuals differently [36]. Negative emotional implications, e.g. self-doubt, shame, guilt, fear, anxiety, and avoidance depend on available coping mechanisms [31, 37]. As such, internalization of negative stereotypes towards oneself, referred to as “self-stigma”, can lead to particularly severe ramifications when individuals start to agree with the contents of society’s negative perceptions [38, 39]. Self-stigma may interfere with identity construction when stigmatizing attitudes manifest themselves in a person’s self-concept and sense of self [30]. Similarly, social stigma exerts significant influence over relational aspects of identity that may cause identity gaps through conflicting social roles [30]. Structural discrimination and social withdrawal often deprive individuals of equal opportunity [40]. Recent contributions to stigma research have highlighted different forms of stigma, such as “stigma by association” or “family stigma”, illustrating that stigmatizing behaviour can be detrimental beyond the scope of the individual it was initially ascribed to [37, 41].

Women or girls who were sexually abused by armed forces and groups face severe prejudice in DRC [42]. In a population-based survey, one-third of Congolese men and women said they would not accept sexual assault survivors back into their communities [43]. Bearing a child from SGBV has been found to impose severe social, health and psychological consequences, leaving victims to feel devalued, hopeless, angry, anxious, or overwhelmed [44]. Due to the fear of stigmatization and inadequate justice and service responses, sexual violence related pregnancies (SVRP) might be aborted, abandoned, and remain largely unreported [44, 45].

It has been illustrated that children deepen the shame experienced by survivors and that communities struggle even more with the acknowledgment of the children conceived from sexual violence than with the reintegration of sexual violence victims themselves [45, 46]. However, the relationship of mothers and children born as a result of SGBV, as well as the impact of stigma on this relationship are not well understood. In 2015, Rouhani et al. assessed parenting attitudes towards children raised from SVRP among a large sample of mothers in the South Kivu province of eastern DRC [47]. The results showed that the majority of the interviewed women had positive feelings towards their offspring, yet two-thirds reported often picturing the assault when looking at their child. The prevalence of perceived stigma attached to the family was nearly 40%. Acceptance by family and community was related to a better maternal-child relationship, while experiences of stigma, maternal anxiety and depression led to lower parenting indexes. Mothers perceived acceptance from the spouse to be the lowest, followed by the community and the family. The study did not account for children’s perceptions of stigma, yet the importance of addressing stigmatizing behavior towards children in DRC is paramount.

Stigma behaviour towards CBOW has been observed in many cultural contexts, including countries with German occupation forces in World War 2 and the Rwandan genocide in 1994 [48]. Conceived during conflict, CBOW are often ascribed multiple stigmas due to overlapping burdens [48, 49], e.g. being born out of wedlock and having an inter-ethnic background. Thus, their experiences with stigma might be particularly threatening to their social identity [24, 26]. Yet, few studies have investigated this group and their participation in society. To our knowledge, no detailed research specific to the life courses of children fathered by UNPK personnel exists. The authors aim to close that knowledge gap through the first transgenerational analysis that addresses the psychosocial situation of PKFC and their mothers. In doing so, we aim to expose the needs of a vulnerable and hard to access population and inform policies for their assistance.

## Methods

### Study design and sample selection

This study is developed out of a wider mixed-methods research project examining relations between UNPK personnel and the local female population in eastern DRC. Within the larger study, community members around six UN bases were asked to share and interpret a story about a girl or woman interacting with peacekeepers. All women who shared first-person narratives about conceiving and giving birth to a PKFC were invited to participate in a follow-up qualitative interview. Snowball sampling was used to increase sample size, with participating mothers of PKFC inviting other women who were raising PKFC, as well as their children to also take part in the study. The current analysis is based exclusively on these follow-up qualitative interviews. All participants were approached face-to-face. Interview locations were chosen according to UN base size, years of operation and nationality of the troop contributing country: Bukavu and Kalemie to the south, and Beni, Bunia, Kisangani, and Goma to the north, representing the major cities with a UN presence in eastern DRC. All interviews were conducted over a 9-week period between June and August 2018.

The larger mixed-methods study was implemented by “the Multidisciplinary Association for Research and Advocacy in the Kivus by United Junior Academics” (MARAKUJA) and by “Solidarité Féminine pour la Paix et le Développement Intégral” (SOFEPADI). MARAKUJA is a non-profit network of Congolese researchers that design, implement, and evaluate large-scale research projects. SOFEPADI is a Congolese non-governmental organization that promotes the rights of women and girls and advocates for their equal access to social justice. MARAKUJA oversaw the logistics of the project. Two female SOFEPADI research assistants supported the mixed-methods data collection in addition to conducting all follow up in-depth interviews. As social workers, both SOFEPADI research assistants had experience working with vulnerable populations, including victims of SGBV. All research assistants were fluent in local languages and completed a five-day training on research ethics, standardized interviewing and data management immediately prior to data collection. Given their cultural, linguistic and content expertise, the project relied on SOFEPADI’s guidance to ensure that the research was designed and implemented in a culturally sensitive manner. SOFEPADI also created a support system for participants in need of psychological counselling and provided referral cards for additional support services at the end of each interview session.

### Survey design and delivery

Semi-structured interview guides were developed with topic questions and a series of prompts, allowing flexibility regarding sensitive issues. The questions were open-ended, and were designed based on the revised literature and core components of stigma proposed by Goffman, and Link and Phelan [29, 33]. Although self-constructed to fit the specific subpopulations, items in the questionnaires drew from existing instruments such as the “inventory of stigmatizing experiences” [50] and its adaptation for CBOW by Kaiser, Kuwert and Glaesmer [51]. The questions were organized into thematic categories and tailored to the participant subgroup to understand the lived realities of both mothers and PKFC. Mothers were asked to provide insight into the family’s demographics, the circumstances surrounding the pregnancy, and the reaction of others to the birth of the PKFC. The survey also asked about exposure to stigma from family and community, and about facets of the mother-child relationship. Depending on their age, PKFC were asked for information about relationships within their immediate social circles and perceived social status. While younger children (aged 6–12) were approached with broad questions that did not mention their heritage, interviews with adolescents (aged 13–19) asked more explicit questions in reference to their heritage. For instance, with young children social status was addressed with questions such as “Some people may think that you are different or more special than other children here, do you know why they would say that?”

Interview guides were written in English, translated to Kiswahili and Lingala, and independently back translated to English by professional translators. The interview questions were refined and tested for comprehensibility and cultural sensitivity by the SOFEPADI research assistants. Any discrepancies were resolved by consensus and a cross-check confirmed that there were no substantive differences in the content being delivered across geographical regions or languages. All interviews were conducted in Kiswahili, or Lingala and were audio-recorded using Zoom H4n Pro devices. Every effort was made to create a safe space for participants that ensured data confidentially and protection.

### Data analysis

Qualitative thematic content analysis was used given the explorative nature of the research. The strategy for analysis was inductive, triangulated with the main themes arising from the literature. The data was open-coded based on emerging themes and overarching narratives, creating categories, sub-categories, and relationships between them [52]. Perceived stigmatization was classified according to its most commonly listed causes and the magnitude of the detailed experiences, based on frequency and impact on participants lives. The codebook was generated by the first author using NVivo V12.2.0 [53] and discussed with the wider research team. The quotes were chosen by contextual relevance.

### Ethics considerations and informed consent

After reviewing the participant information and consent forms in Kiswahili or Lingala, participants gave verbal informed consent. PKFC were provided with this information in an age appropriate manner under the prerequisite of parental consent. Verbal consent was recorded, and data confidentiality guaranteed. Written consent was waived due to possible illiteracy and the minimal risks involved. The degree of sensitivity inherent in the broad and open questioning of young respondents enabled their inclusion in an ethical manner. No personally identifying information was gathered, and all interviews took place in private locations to mitigate potential harm resulting from being singled out for an interview. Participants were not paid to participate but they were provided with light refreshments at the time of the interview. If an interview had to be arranged for a later time, mobile phone credit was offered to facilitate scheduling. Participants had an opportunity to ask questions and were explicitly told about their right to refuse the interview or withdraw from the project. The research was approved by the University of Birmingham’s Ethical Review Board ERN_18-0083 (or protocol 18-0083) and by the Queen’s University Health Sciences and Affiliated Teaching Hospitals Research Ethics Board (protocol 6,019,042).

## Results

### Study population

A total of 95 individual interviews (35 PKFC and 60 mothers) were conducted. Children in the age group 6–12 (*n* = 22) were on average 8.4 years old with a Standard Deviation (SD) of 3.6 years at the time of the interview. Adolescents in the age group 13–19 (*n* = 13) were on average 14 (SD = 1.8) years old when the study took place. Among mothers who provided details on the nature of the sexual interaction (*n* = 53), 37 mothers (69.8%) acknowledged that it was consensual, oftentimes involving romantic ideas and expectations. Ten women (18.9%) described the relationship as being predominantly transactional in nature, centred around the exchange of food, money, clothing or other items in return for sex. However, transactional and romantic relationships were not clearly distinct or mutually exclusive, and most consensual relationships included presents and material expressions of affection due to the adverse socio-economic conditions in DRC (see [54] for categorization of sexual interactions in the context of UNPK). Six mothers (11.3%) reported becoming pregnant after being raped by one or more peacekeepers. At the time of the interview, mothers were on average 26.2 (SD = 7.2) years old; compared to an average of 19.7 (SD = 5.9) years at conception. The youngest girl reportedly impregnated was 10 years old and the oldest woman impregnated was 36 years old, with 46% of the mothers being under the age of 18 when they conceived. In the current data set, 75% of mothers (*n* = 45) recalled the nationality of the father precisely, although most used the terms “black” and “white” for descriptive purposes. The most commonly reported countries of origin for peacekeeper fathers were: Tanzania (37.7%), South Africa (15.6%), Morocco (6.7%), Uruguay (6.7%), Benin (6.7%), Malawi (6.7%), Sudan (4.4%); Senegal (4.4%), Guatemala (2.2%), DRC (2.2%), Bangladesh (2.2%) and Nepal (2.2%).

### Stigma experiences

The analysis uncovered the often-challenging relationships of mothers and PKFC with their families and communities*.* Stigmatization was assessed based on individuals facing labelling, stereotyping, separation, status loss and/or discrimination [33]. The magnitude of stigma was evaluated through consideration of frequency and impact of the shared experiences. Perceived stigma by PKFC was compared to the observations of their mothers who were asked to evaluate their own and their children’s social interactions.

Of the PKFC surveyed, a majority articulated stigma. Children aged 6–12 years were not asked explicit questions about acceptance and were therefore less likely than adolescents to comment on their integration. For both age groups, stigma was manifested in a range of experiences; from teasing and bullying to overt discrimination, abuse and neglect. As demonstrated below, participants detailed severe cases of physical and verbal abuse, which in some instances made it difficult for PKFC to form meaningful relationships.

*“They strike him a lot at home. They insult him, they make fun of him, scorn him and don’t take care of him much, sometimes they even beat him. His nickname is ‘Aramu’ [Motherfuck]. He feels rejected and very sad when they behave like that.”* (Mother, Goma).

Stigmatizing attitudes and behaviour were also a reality for the mothers. The deterioration in social status after giving birth to a PKFC was the most dominant and recurring theme in interviews with mothers. Many participants reported an altered reputation and frequent issues with connectedness and closeness to friends, neighbours, and other members of the community. More than two thirds of mothers reported facing far-reaching consequences of stigmatization, with several being cut off entirely from family ties and resources. The most severe forms of stigmatization included becoming the subject of gossip and harassment in the community to a point where mothers felt the need to relocate.

*“It affected me a lot. Not only did he leave me and the child, but the information was spread in my community. I am no longer respected there. A child outside marriage makes one suffer, especially when it gets ill and the parents have chased one away from home. I get embarrassed to ask for help. I feel bad and like my life is sinking, I am regretful.”* (Mother, Goma).

**Fig. 1:**
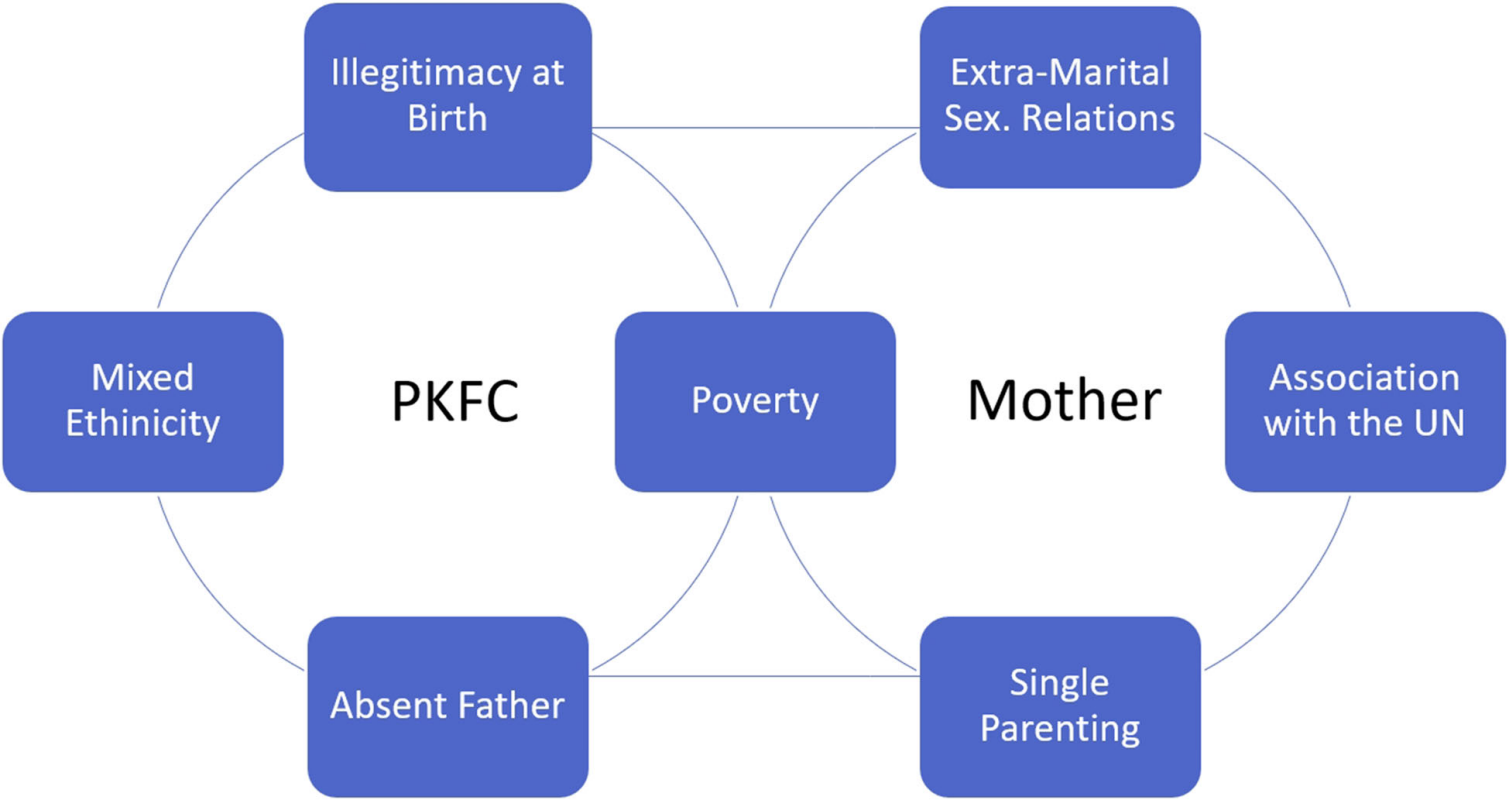
Dominant reasons for perceived stigmatization

Figure 1 illustrates the most frequently listed reasons for perceived stigmatization. Each of the contributors to stigma will be explored in greater detail below. Overlapping content due to parallels in the experiences of mothers and children will be combined under joint headlines and discussed in light of transgenerational influences. As such, the feeling of illegitimacy among children is illustrated against the backdrop of their mother’s extra-marital sexual relations. This is summarized under the heading context of conception. Stigma based on fatherlessness and single parenting will be debated in light of local norms regarding family structure. Lastly, othering of PKFC related to mixed ethnicity will be discussed in conjunction with their mothers’ connection to UNPK under the headline of ethnic belonging.

### Poverty

A large proportion of the population in eastern DRC are impoverished and gendered socio-economic disparities are widespread [55]. Having a child out of wedlock adds significantly to these pre-existing financial insecurities, especially for women who tend to work in lower paid, informal sectors. Besides having to cover the expenses of pregnancy and maternity, mothers reported that raising a PKFC was a barrier to marriage as well as economic and educational activities which could have provided some degree of financial stability.

*“Some say I am not intelligent; I should have aborted: ‘your schoolmates are completing their studies while you must stay home and raise a child by yourself’. These thoughts bring me much suffering and raise doubts. I wanted to become a teacher.”* (Mother, Kalemie).

Every fifth participant in our study pointed towards a shortage in resources as the main reason for their social stigmatization. The example below reflects the situation of a 16-year-old mother who was exploited through her family and forced into child prostitution as a result of poverty. Although impregnated by UN personnel at the age of ten, this young mother names her lack of income as the dominant reason for social exclusion.

*“My family cannot support me because they have no means, they are destitute. I think if people treat me different, it’s because I’m jobless and I have no activity that generates income like a small business… Currently we are starving.”* (Mother, Goma).

After giving birth to a PKFC, women were almost without exception left in a situation of financial hardship. Mothers in our study received negligible assistance from the individual peacekeepers. Yet, there appeared to be a hierarchy amongst them, with women who had longer-term support from the UN peacekeeper at the top and other women being looked down upon for not securing similar benefits.

*“Many mock me and laugh at me. Some do this because unlike me they were lucky enough to get money, plots of land or houses from their MONUSCO boyfriends. They say I am miserable and cursed for not having been offered such things by my South-African husband.”* (Mother, Bukavu).

Poverty also played into the stigma experiences of PKFC. Children and adolescents in the current sample described lack of shelter, food, and other basic resources, as well as the inability to afford health care and schooling. Younger children exemplified sparse economic means by not possessing certain toys and thus, feeling left out during playtime.

*“My friends sometimes decry me by saying that I use their dolls while never bringing any of my own. This makes me feel sad and upset and triggers thinking of my departed father. They sometimes insult me by saying that I am crazy, stupid, imbecile and poor. They insult me this way because of poverty.”* (PKFC, 12, Bukavu).

The longing for financial security was usually discussed in the context of the unaccountable father and the aggravated economic conditions caused by his absence. Observing PKFC’s deprivation of a decent living standard created additional pressure on mothers.

*“My children are different from other children in that they are dressed poorly; you see it when they meet for birthday parties. When others are dressing up, they complain because their peers’ fathers bought them brand new clothes and shoes. It’s heart-breaking to experience such situations. They are being told that they are street children who have no father.”* (Mother, Goma).

Some women found themselves in a downward spiral of further social rejection when extreme poverty led them to (re) engage in sex-work to meet their child’s basic needs.

*“I didn’t care what was right or wrong. I was willing to do whatever in return for food … My worries were to find clothes for the child. I was living far from my family, I stopped going to school and willingly became a prostitute. Men came to sleep with me and gave me money which helped me a lot.”* (Mother, Goma).

### Family structure

Verbal attacks and harassment related to family structure were described by both mothers and children. Participants fathered and abandoned by peacekeepers experienced stigma as a result of their absent biological fathers and paternal families. Not knowing the identity and/or whereabouts of parents and growing up without the social support of extended families were among the most commonly cited reasons by PKFC for being stigmatized.

*“Some people say I am different because my father left when I was a baby. Some people are amazed to see I am still alive.”* (PKFC, 14, Bukavu).

Comments about fatherlessness were frequently linked to the absence of a male role model and head of household. Teasing or bullying by classmates also projected prejudice against peacekeepers on to the children and portrayed them as conceited, privileged or violent.

*“I really feel sad when I realize that my child isn’t treated fairly. If his father was here, he wouldn’t undergo this kind of discrimination. Others sometimes call him bad names, like ‘son of a hooligan’, ‘son of a dumb father’. It makes me indignant!”* (Mother, Bunia).

A large majority of PKFC reiterated being told to “go to their fathers” or “follow their dads” in order to find relief from familial and socio-economic hardship. These comments support the representation of PKFC as an “out-group” in their community, deprive them of a sense of belonging, and propagate the idea of an incomplete identity.

*“My comrades mock me by saying that I have no father. They hurt my heart when they say that I have no shoes or clothes. When I reply that there is no one to support me, they ask me why I can’t follow my dad.”* (PKFC, 9, Goma).

The perception of fatherlessness as the reason for hardship was reported by PKFC of all age groups, including the following young adult born in 1999 when UN peacekeepers first arrived in DRC. The emotional and physical abuse driven by his siblings distanced the 20-year old from his own home and denied him safety and shelter.

*“It’s my stepbrother and sister who treat me differently. It has happened at times that my brother came home late drunk and found me on the bed sleeping and started disturbing me, chasing me away from the house. In these situations, I would sometimes spend the night outside. Other times my mother intervened so that I can go back to the bedroom. If he is really drunk, he beats me up. Recently he cut my mat into two parts. If I was with my father such discrimination wouldn’t exist, I could be happy like other people.”* (PKFC, 20, Kalemie).

Ostracization of the mothers partially resulted from being a single parent. Tainted as a violation of family code and social norms, this theme paralleled the theme of “fatherlessness” in its expression of familial hardship. The socio-economic burden of raising a child without a male partner, and raising a PKFC in particular, often rendered women unmarriable and hence lowered their social status in the eyes of the community. Several mothers spoke about their lowered prospects of new relationships; few mentioned long-term partners.

*“Because of this child, my life is dark, no men will ever intend to marry me. I am 36 years old. My life is bound to difficulties.”* (Mother, Kalemie).

In Congolese culture, husbands do not necessarily provide for children from earlier relationships. Hence, instead of improving the socioeconomic situation for PKFC, new partners sometimes caused more severe rejection and increased the family’s dysfunction.

*“What troubles me the most is that my husband doesn’t want to pay the school fees for my daughter. Moreover, he insults her a lot. I must wonder about the future of my child.”* (Mother, Bukavu).

### Ethnic belonging

A number of PKFC listed “mixed ethnicity” as the predominant cause for stigma. Children reported being humiliated or ridiculed on the grounds of not being Congolese, being “white” or “foreign”, or otherwise singled out for their ethnic heritage. They also recalled negative stereotyping attached to not being able to trace their family clan or ethnic group.

*“The child is rejected by their family; they are not accepted amongst them. They tell the kid that he is a foreigner. Children bully him to leave and go to South Africa, his father’s country. They rebuke him for not being Congolese. When he is playing with others, they tell him all kinds of insults. Most often to go back to his country. They regard him as a foreigner, just as his father.”* (Mother, Bukavu).

Related to their inability to trace their origin, PKFC were affected in their identity creation and suffered from isolation. The country of the father’s origin or the children’s skin colour was frequently used as a derogatory name for PKFC; participants were referred to as “Nepali”, “Tanzanian” etc. or “White” instead of addressed by their first names. Thus, stigma heightened their racial awareness and left them questioning their cultural identity.

*“Sometimes, he feels anxious. Especially when other children make fun of him and call him Tanzanian. He cries when they shout, ‘get out of here, Tanzanian’.”* (Mother, Beni).

Cases of social marginalization and maltreatment appeared more severe for mixed-race children who clearly stood out as biracial. The data revealed that experiences of stigma occurred more often and more persistently for PKFC fathered by “white” internationals.

*“The neighbours don’t love her. They constantly make fun of her saying that she is the ‘daughter of a white’. My friends were even bringing different kinds of poison in order to kill her. Because of this she only stays here in the compound, she is ashamed.”* (Mother, Bukavu).

In line with this, children seemed to struggle less with stigma if their non-Congolese paternal lineage was not as clearly visible and if they were still considered “black”. In anticipation of negative reactions, some women decided to refrain from disclosing the ethnic heritage of their children in order to avert stigmatization and adverse life experiences.

*“The kid doesn’t know anything about his father, I am the only one who knows his secret. I always lie; tell him that his father lives here in the area, although he is from South Africa and I don’t know where he is. And still, the child is often discriminated by others*.” (Mother, Kalemie).

When non-disclosure as a strategy of protection failed, PKFC and mothers were confronted with their “otherness”. For mothers, being linked to UNPK was anticipated to be a factor for losing the status of a “respectable” woman. Mothers described the damage to their reputation to be particularly severe based on a perceived “association with the UN”.

*“My family blames me and say they won’t take care of one of their children, I shouldn’t have gone to work for someone like that and I shouldn’t have gotten pregnant. It makes me very sad.”* (Mother, Goma).

We found a consensus that relations with UNPK personnel impaired prospects for future relationships, more so than extra-marital intimate relations with local men. The data does not capture the sentiment of the general population towards peacekeepers; therefore, it is not clear whether this reflects negative attitudes towards the work of the UN specifically or relationships with non-Congolese men more generally. Yet cultural expectations and family code seemed to oppose relationships with UNPK staff.

*“They call me a Beninese’s wife. My reputation is ruined … When people learn that you were once friends with a guy from MONUSCO they start despising you and talking ill of you. It is not easy to find another boy or man-friend if you have been deceived by one of them.”* (Mother, Kalemie).

### Context of conception

An equally large group of PKFC comprehended the stigma directed at them to be linked to their mother’s place in society rather than their father’s background or ethnicity. These participants felt singled out because of their mothers’ lifestyle or behaviour. Some of the most recurrent insults directed towards PKFC were “daughter/son of a bitch”, “bastard” or “illegitimate”, portraying the children as the products of rape, sex-work, or a parental relationship that otherwise conflicted with social norms. This has been summarized under the theme of “illegitimacy at birth”.

*“The child is discriminated everywhere he goes. He is insulted as a ‘son of a bitch’. They say he isn’t worth it to be alive.”* (Mother, Kisangani).

Since the illegitimacy of children was understood to be rooted in the “context of conception”, this theme naturally emerged in mother’s interviews. Due to cultural and religious values as well as restrictive abortion laws, some women felt torn between having a child out of wedlock versus undergoing an abortion. Despite not being explicitly asked about the decision-making process, a small number of women discussed how they would be stigmatized if they kept the pregnancy, while others, reported that they would be stigmatized if they aborted the pregnancy.

*“So many people told me to abort so I wouldn’t be called the mother of a MONUSCO child. They said I would never find another one to love me if I kept the pregnancy.”* (Mother, Kalemie).

*“I thought about aborting the baby but when I asked other people for advice, they made it very clear that it is shameful to commit such a crime. I insisted at first, contending that I might give birth to a ‘mulatto’, but they convinced me that this wouldn’t be a problem; at least not if MONUSCO was still in the area.”* (Mother, Bukavu).

Due to the local understanding of fidelity and marriage, women who are known to have had “extra-marital sexual relations” struggle to maintain their societal status. Exposed by pregnancy and childbirth, mothers faced prejudice for being perceived as promiscuous and disrespecting traditional gender roles; often regardless of whether the sex was consensual. One quarter of mothers reported that their pregnancies occurred due to transactional sex, sex-work, rape or gang rape. Yet, women often bear the blame for these incidents. One participant who reported a gang rape by MONUSCO personnel was heavily stigmatized after being impregnated at the age of 13.

*“People started wondering where this little girl got her pregnancy from. They laughed so much at me. They said look at her who has been raped, she has a white child. Many people laughed at me. I felt so insulted, all of this hurt me so much.”* (Mother, Beni).

Despite rape being outside of the victim’s control and women almost exclusively engaging in transactional relationships out of necessity, stigma was particularly severe for this group. They were presumed to be HIV positive and were socially tainted for not being able to identify their children’s fathers.

*“What do people say? They often just stare at us and then gossip about our personality. They contend that we are the sex chattel of South Africa. They mock and target us, wherever we go. They accuse us of aids etc. I have no power really. I simply sigh and take the blame. I have cried and cried over this. God is my last resort.”* (Mother, Bukavu).

### Intergenerational aspects of stigma

Reasons set out for perceived stigma and discrimination were often two or threefold and most individuals carried a combination of labels. Inherent in many of these narratives were experiences of stigma that resided in the mother-child relationship and their social status as a unit, which will be the subject of this section.

*“All my adventures were secret until the pregnancy revealed them and since they discovered what I have done, they don’t love me anymore. My child is often scorned because he is a MONUSCO bastard and a foreigner. He is discriminated and insulted, and I am being blamed for it.”* (Mother, Bukavu).

This snapshot of a young mother’s life signifies that the stigma experiences of mother and child interact. Moreover, it gives a brief insight into negotiations of guilt and responsibility. While children occasionally understood their stigma to reside in the nature of their conception and their mother’s social circumstances, mothers reported that the existence and social exclusion of their children were aggravating to their own social standing.

*“Some people also tell me that I am scolded because of my child … but she is my birth daughter, my flesh and blood. Nobody can reject their birth child, even if they were ugly.”* (Mother, Goma).

The succeeding generation’s victimization led to additional stigma in mothers, that was based on being a neglectful parent. Not being able to provide for children and not having secure and stable livelihoods was socially perceived as personal failure. Many mothers reported feeling responsible for their children’s difficult socio-economic circumstances, like they were passing their own hardship down to their children. In that sense, the fractured social identity of PKFC increased mother’s problems with integration and acceptance.

*“A child without a father is called ‘Shanga’ in North Kivu. He is not accepted. He is stigmatized because I’m stupid, that’s what they say. It makes me sad. He laughs with others, even with me sometimes but he also gets told: ‘You, a child without a father, or you, a child without an aunt in Congo’. He feels internal sadness. When I see my child, I feel unhappy. I’d like to build a house for his welfare.”* (Mother, Goma).

In a number of cases, these dynamics had a bearing on the mother-child relationship and adversely affected the bond between PKFC and their mothers. Dysfunctional relationship patterns not only reinforced community stigma, as outlined above, but also increased the likelihood for mothers and children to internalize aspects of stigmatization. For instance, a proportion of participants experienced mood alternations and showed symptoms of depression and suicidal ideation. This was particularly true when painful recollections about the circumstances of conception or, difficulties linked to giving birth, evoked conflicting emotions in mothers such as: love, hate, shame, hope or desire to abandon the PKFC. As a result of opposing feelings, disturbances in the attachment of mothers and PKFC were observed.

*“People in the community gossip much about my life. Some say that I broke my marriage promise because of a foreigner who is the reason I am now a desperately poor woman. It hurts me so much, but I have no other choice as to stand it. I sometimes wonder whether I should kill myself or my child, but I guess I just need to hold on and bear the consequences of my decisions. If his father returns, we can get our dignity back.”* (Mother, Bunia).

While some PKFC reported having a good relationship with their mothers, others experienced similarly ambivalent or negative emotions towards them. A handful of PKFC gave accounts of child maltreatment or reported that their mothers were physically or psychologically unable to care adequately for them, with the latter sometimes raising questions for the PKFC about their right to exist. One respondent detailed that being deprived of motherly care left her feeling worthless and not deserving of love.

*“Oh, she never cares about her child - me. She only roves, roams and wanders in the city all day long, and when she comes back home very late at night or in the evening, she meets me and starts shouting at me. She never talks to me in a friendly way. She says I have no value at all for I’m not like her other children. When she says that, I feel like it’s better to take a knife, stab myself and die once and for all. I feel very upset for I have no support from my relatives, apart from my grandparents. Other children who are living with their parents must be living well, I think.”* (PKFC, 13, Bukavu).

Children might absorb their mothers’ experiences of discrimination and feel like a burden that has caused them misery.

*“People sometimes challenge me by saying that I am not Congolese. That offends me. They also say that my mother became pregnant because of her prostitution. She keeps feeling sorry for that. It disturbs and hurts me so much …*. *Sometimes, I ask my mother why she decided to give birth to me.”* (PKFC, 13, Kisangani).

## Discussion

This qualitative study, including 90 in-depth interviews, explores the spectrum of stigma experiences as reported by PKFC and their mothers in eastern DRC. Little empirical data currently exists on the situation of children born to UNPK beneficiaries in host countries. The current study narrows this knowledge gap through perspectives on family and community integration. Data collection showed that the identity of PKFC was well known and that even the youngest participants were aware of stereotypes that originated from the circumstances of their conception. Our results suggest that mothers and children received negligible assistance from peacekeeper fathers, leaving them almost exclusively in a situation of financial hardship. Despite a range of experiences, both mothers and children were found to be situated between acceptance and rejection. The data revealed four key areas causing stigma. In addition to these, we identified similarities between drivers of stigmatization that suggest intergenerational aspects and stigma transmission between mothers and children.

The first and most consistent cause of stigma, “poverty”, was identified to have a large influence on the acceptance of mothers and PKFC in their community. Confirming prior literature [11, 14, 54], the adverse pre-existing socio-economic conditions in DRC were often found to be a reason for mothers to engage in sexual relations with peacekeepers. The demographic profile of mothers in our study indicated that pregnancy often occurred in young women and those from poor backgrounds. Contrary to the initial hopes voiced by mothers, having a PKFC rarely led to an elevated social status but was intertwined with more severe lack of resources and opportunity. Reportedly, the social status of families was related to the availability of financial means; for instance, mothers and children mentioned that the inability to cover school fees had an impact on their reputation in communities and raised questions about child support. Giving birth to a PKFC often complicated family relations and prevented young mothers from continuing their own education, significantly limiting their prospects for employment. Mothers detailed being looked down upon for not securing alimony payments or other benefits from former partners. Stigmatization of individuals with conflict-related trauma has previously been described to be exacerbated by contextual factors such as economic deprivation [56]. In the present study, stigmatization did not occur universally but was mediated by different factors that increased participants’ vulnerability, such as socio-economic hardship. Accordingly, the social challenges of families with PKFC were reinforced by low financial security, a condition that worsened when family support was withdrawn. Consequently, having a PKFC did not only lead to immediate alterations in the social status of mothers but set off a chain reaction that reinforced stigma and status loss in the long-term.

Secondly, family structure and prejudice centred around the “absent father” or life partner were reported to be factors contributing to the stigma experiences of participants. Growing up without a father in a patrilineal society with clear gender roles, PKFC faced challenges integrating into their communities and developing a cultural identity that was accepted by their peers. They described the longing for a “normal” family life, the lack of a male role model and support from both parents to be the source of stigma. Like PKFC, mothers sensed that being a “single parent” and raising a PKFC by themselves prevented them from entering new partnerships and building families that complied with social norms. Confirming theoretical assumptions [57, 58], this indicates that a less conventional family unit provoked negative attention and demoted the social status of mothers and children. Replicating what is known from CBOW in other settings [49, 59, 60], we found that stepfathers did not accept children from previous relationships, which turned reconstituted families into an additional factor for rejection. In instances where preconceptions about the circumstances of conception drove kin networks away, PKFC did not grow up in traditional compounds. For communal societies in which extended family is at the centre of social activities, being deprived of this integral part of life is severely problematic [61]. Moreover, it can be expected that less traditional living arrangements worsened socio-economic challenges, like household income, reinforcing poverty and the stigma attached to it.

Thirdly, both groups mentioned stigma attached to issues surrounding ethnic and cultural belonging. This was tangible for PKFC when being taunted for their “mixed-ethnicity” or stigmatized on the grounds of not being Congolese, being “white” or foreign. The uncertainty regarding the origin of children’s inter-ethnic appearance was perceived to amplify their outsider status. Physical features that evidently identified PKFC with their father’s ethnicity made them an easier target for stigmatization and conveyed more potential for societal rejection. In line with this, mothers mentioned “association with the UN” to be the root of a loss of status as well as familial and communal problems. Having a child visibly connected to a foreign army has been shown to cause stigmatization and considerable hardship in other settings [62, 63]. Our study shows that being associated with non-Congolese paternal lineage distinguishes families in eastern DRC from the general population. Stereotypes towards relationships with internationals or those working for international organizations seemed to have enabled individual or structural discrimination against women in their communities. The complexities of social processes nourishing these prejudices and whether they are UN-specific remains to be explored.

Lastly, each group signified stigma based on the circumstances surrounding conception. PKFC noted social repercussions for being born “illegitimately”, while mothers pointed towards the local understanding of marriage and fidelity to explain their altered social status after “extra-marital sexual relations”. The data illuminates a range of experiences regarding the nature of conception, from consensual, romantic relationships to rape with extreme violence. Previous research in contexts where the value of women is traditionally defined by reproductive exclusivity has shown that sexual violence can lead to social ramifications as severe as the attack itself [64, 65]. In line with earlier work [59], the present study suggests that rape and sex-work particularly diminished acceptance for mothers and children. However, more quantitative studies with higher sample sizes are needed to confirm such observations. Approximately half of the mothers interviewed were minors at the time of conception, including a girl impregnated at the age of ten and several girls impregnated at the ages of 13 and 14. We found that independent of consent and age at impregnation, mothers reported a community perception that they were responsible for the events leading to conception. While PKFC were often confronted with being the product of inappropriate sexual relations, mothers reflected on stigma attached to raising a child born out of wedlock. Hence, both were repeatedly reminded of the contextual factors leading to conception and the “illegitimacy” of their actions / existence.

The social judgment associated with the themes explored in this study affected the reality of both groups collectively. Based on parallels in the experiences of mothers and children, we argue that each source entails elements of transgenerational stigma. Stigma ascribed to mothers was passed down to children via two channels or modes of transmission: directly, when communities punished PKFC for their mother’s experiences, or indirectly, moderated by the mother-child relationship. Evidence suggests that interpersonal trauma like sexual abuse has noticeable negative impact on post-partum bonding [66]. Thus, the risk of a weak maternal bond is elevated for PKFC conceived through rape since mother-child attachment may be influenced by their mother’s traumatic experiences. Social marginalization, especially when internalized, was detrimental to mothers’ abilities to provide care and stability for their children. Earlier work has shown that insecure attachment and a compromised relationship between caregiver and community, significantly affects the development and maintenance of child mental health [67–69]. In the present study, being neglected by a parent reinforced negative social interactions and internalizing symptoms for children. Conversely, PKFC’s experiences with social hardship and community stigma have also been shown to influence mothers. Earlier research in eastern DRC examined the mental health of women raising children from SVRP and found that stigmatized children significantly increased the likelihood for most mental health disorders in mothers [70]. Similarly, prejudices directed at PKFC transferred to mothers when the unfair treatment of their children provoked low mood in caregivers. Hence, the social standing of PKFC and their mothers are interrelated and experiences with social marginalization have proven to be a salient factor driving the mother-child relationship. Longitudinal research targeting the social status of mothers and children over time will be essential to detangle how their situations affect one another.

### Limitations

The sample in the present study is not representative. Thus, the results cannot fully be generalized to PKFC in the DRC or other countries hosting PKO. Participating in an interview with an international research team might have raised expectations regarding long-term outcomes of the research. As such, it is conceivable that participants may have overreported issues around poverty to secure personal benefits or described their situation as less severe to avoid further societal repercussions. For participants with children conceived during the MONUC era, pregnancy and childbirth were more than 10 years in the past and the events might not be reliably recalled [71]. However, the focus of the present analysis is on stigma which persisted to the time of data collection. Due to the sensitive nature of the research and different age groups being accommodated with unique survey instruments, questions were sometimes asked inconsistently which resulted in occasional missing data. Lastly, the analysis was conducted by a group of non-Congolese academics with inherent bias regarding cultural norms. Although local experts, researchers and translators were consulted along the way, nuances of the narratives might not always have been appreciated fully.

### Contributions

The limitations above notwithstanding, the present study makes several notable contributions. Being the first to provide transgenerational empirical data on CBOW, the results are expected to be highly relevant to conflict-affected youth in a variety of contexts. Research in different settings suggests that stigmatization and discrimination represent formative experiences in the childhood and adolescence of many CBOW, regardless of their paternal background [25, 72]. Previous insights into the stigma of German Occupation Children born in the aftermath of World War 2 identified the “father’s origin”, “physical attributes” and “illegitimacy at birth” as anticipated causes for stigma [23]. In the present study children also listed fatherlessness, poverty and transgenerational elements as precursors for stigmatizing behaviour. It seems likely that for children in the current sample, the familial and social burdens faced by CBOW in other settings are exacerbated by the adverse socio-economic conditions in DRC. The extreme level of poverty, combined with issues of race and neglect caused intersectional stigma and impacted children’s self-concept, social and cultural identity. Moreover, the experiences of stigma not only caused difficulties in constructing coherent and positive identity narratives but also resulted out of PKFC’s difficulties to manage their multiple identities aspects. This echoes the experiences of CBOW in other settings [23, 24, 26].

Moreover, we show the importance of prioritizing intergenerational aspects of stigma for CBOW. Few studies to date have discussed links between maternal and child mental health in conflict-affected settings [67–69]. Yet, the present study makes a connection between the two and suggests that their social standing is intertwined. It is thus, all the more important to consider difficulties that emerge for CBOW within the context of their family and community.

The current study advances existing literature on sexual misconduct during PKO by adding empirical data on the individual experiences of affected women and children. It is unique in that it provides the largest sample to date of qualitative interviews with women who have conceived children with UNPK personnel and illuminates children’s struggles through their own thoughts and emotions. In conjunction with the recently published studies on MINUSTAH [14, 54], it addresses the gap in literature on CBOW in the UNPK context. By including rich qualitative data, we take the narrative-based approach of Lee and Bartels further, and contribute significant information to their evaluation of community perceptions. While not being exhaustive in its exploration, the study demonstrates the personal experiences of two generations by directly voicing the concerns of a highly vulnerable population. To our knowledge, this is the first study to include children’s perspectives in a systematic analysis of sexual misconduct during UNPK. PKFC are considered a hard to access population group and interviewing them carries a variety of ethical challenges [72, 73]. Yet, not including them in research that concerns them negates the possibility of them being active partners and authorities in their own right. Thus, we believed the benefits of their participation to outweigh the risks of their inclusion. Our study successfully included them in the research process and documented their social identities by empowering them as social agents. As such, our research shifts the focus of discussions regarding SGBV in humanitarian settings to a new population and contributes to a newly established data base.

## Conclusion

This is the first study to capture transgenerational aspects of stigma by examining the life stories of mothers and CBOW in any geopolitical setting. Previous research indicates that the psychosocial consequences of being a CBOW include a higher risk of growing up in a hostile social environment [22]. We have investigated the assumption that the societal attitude towards PKFC, as a subgroup of CBOW, is conspicuous in stigmatizing behaviour. The context of family and community helped to conceptualize the stigma experiences of PKFC and added an additional layer of understanding to the analysis. The majority of participants reported struggling with social alienation. The core insights regarding the roots of perceived stigma were found to be based upon the following factors:

- Economic factor: Poverty
- Familial factor: Family structure
- Geopolitical factor: Ethnic belonging
- Sociocultural factor: Context of conception
- Intergenerational factor: Mother-child relationship

These factors highlight the web of contextual determinators that represent the background for applied prejudices. While some themes arose consistently and indicate the existence of main influencers, the diversity of personal accounts hints at an interplay of these factors. More work disclosing drivers of rejection and acceptance is needed, yet our research shows that PKFC are a socially disadvantaged group in (post)-conflict settings.

As a result of the data presented, several changes in policy and programming are recommended. First and most important, poverty as the main reason for stigmatization needs to be addressed. Second, psychosocial services tailored to support families with PKFC are necessary so that healthy relationships can be formed, and self-stigma reduced. In addition, preventive measures that lower the likelihood for sexual misconduct and stigma should continue to be implemented. This includes training for UNPK personnel to facilitate an understanding of the vulnerability of local populations in host countries, as well as community-based approaches around reproductive and sexual education for women and girls that increase awareness regarding the potential implications of sexual relations with members of UNPK forces and reduce the stigma attached to it.

Currently, the multi layered legal landscape largely prevents mothers from holding individual peacekeepers accountable and securing child support [9, 74, 75]. Our findings challenge the international community to rethink current policies to effectively benefit the needs of those addressed in this study. The authors suggest that in order to facilitate integration of PKFC effectively, policy and programming recommendations should consider mothers and children as core addressees of assistance and develop services that support them individually and collectively. The present article aims to inform such services; however, the complexities of assistance and reparations will be discussed in forthcoming publications.

## Data Availability

The datasets used during the current study are available from the corresponding author on reasonable request.

## Abbreviations

CBOW: Children Born of War
DRC: Democratic Republic of Congo
MARAKUJA: Multidisciplinary Association for Research and Advocacy in the Kivus by United Junior Academics
MINUSTAH: Mission des Nations Unies pour la stabilisation en Haïti (United Nations Stabilization Mission in Haiti)
MONUC: Mission de l’Organisation des Nations Unies en République Démocratique du Congo (United Nations Organization Mission in the Democratic Republic of Congo)
MONUSCO: Mission de l’Organisation des Nations Unies pour la Stabilisation en République Démocratique du Congo (United Nations Organization Stabilization Mission in the Democratic Republic of Congo)
PKFC: Child fathered by a member of a UN peacekeeping force and born to a local mother in the country of deployment
PKO: Peacekeeping Operation
SD: Standard Deviation
SGBV: Sexual and Gender-Based Violence
SOFEPADI: Solidarité Féminine pour la Paix et le Développement Intégral
SVRP: Sexual Violence Related Pregnancies
UN: United Nations
UNPK: United Nations Peacekeeping
UNSC: United Nations Security Council
